# Validating estimates of prevalence of non-communicable diseases based on household surveys: the symptomatic diagnosis study

**DOI:** 10.1186/s12916-014-0245-8

**Published:** 2015-01-26

**Authors:** Spencer L James, Minerva Romero, Dolores Ramírez-Villalobos, Sara Gómez, Kelsey Pierce, Abraham Flaxman, Peter Serina, Andrea Stewart, Christopher JL Murray, Emmanuela Gakidou, Rafael Lozano, Bernardo Hernandez

**Affiliations:** Institute for Health Metrics and Evaluation, University of Washington, 2301 Fifth Ave., Suite 600, Seattle, WA 98121 USA; Geisel School of Medicine at Dartmouth, Dartmouth College. 1 Rope Ferry Road, Hanover, NH 03755-1404 USA; Center for Population Health Research, National Institute of Public Health, Av. Universidad 655. Col. Santa María Ahuacatitlán, Cuernavaca, Morelos 62508 Mexico; Center for Health Systems Research, National Institute of Public Healt. Av. Universidad 655. Col. Santa María Ahuacatitlán, Cuernavaca, Morelos 62508 Mexico; Ipas, P.O. Box 9990, Chapel Hill, NC, 27515 USA

**Keywords:** Automated methods, Mexico, Non-communicable diseases, Non-communicable diseases prevalence, Questionnaire

## Abstract

**Background:**

Easy-to-collect epidemiological information is critical for the more accurate estimation of the prevalence and burden of different non-communicable diseases around the world. Current measurement is restricted by limitations in existing measurement systems in the developing world and the lack of biometry tests for non-communicable diseases. Diagnosis based on self-reported signs and symptoms (“Symptomatic Diagnosis,” or SD) analyzed with computer-based algorithms may be a promising method for collecting timely and reliable information on non-communicable disease prevalence. The objective of this study was to develop and assess the performance of a symptom-based questionnaire to estimate prevalence of non-communicable diseases in low-resource areas.

**Methods:**

As part of the Population Health Metrics Research Consortium study, we collected 1,379 questionnaires in Mexico from individuals who suffered from a non-communicable disease that had been diagnosed with gold standard diagnostic criteria or individuals who did not suffer from any of the 10 target conditions. To make the diagnosis of non-communicable diseases, we selected the Tariff method, a technique developed for verbal autopsy cause of death calculation. We assessed the performance of this instrument and analytical techniques at the individual and population levels.

**Results:**

The questionnaire revealed that the information on health care experience retrieved achieved 66.1% (95% uncertainty interval [UI], 65.6–66.5%) chance corrected concordance with true diagnosis of non-communicable diseases using health care experience and 0.826 (95% UI, 0.818–0.834) accuracy in its ability to calculate fractions of different causes. SD is also capable of outperforming the current estimation techniques for conditions estimated by questionnaire-based methods.

**Conclusions:**

SD is a viable method for producing estimates of the prevalence of non-communicable diseases in areas with low health information infrastructure. This technology can provide higher-resolution prevalence data, more flexible data collection, and potentially individual diagnoses for certain conditions.

**Electronic supplementary material:**

The online version of this article (doi:10.1186/s12916-014-0245-8) contains supplementary material, which is available to authorized users.

## Background

Non-communicable diseases (NCDs) form a substantial part of the global burden of disease in both developing and developed countries, with certain NCDs posing an equal or greater threat in low-income compared to high-income countries [1]. Mexico, and much of Latin America in general, have seen a relative increase in their NCD burden in the past 20 years, with conditions such as heart disease, arthritis, and vision loss steadily increasing in terms of disability-adjusted life years [1].

Despite the substantial burden of NCDs around the world, it continues to be difficult to collect accurate information on their prevalence, particularly in areas that lack consistent or accessible health care. In part, this is due to inherent limitations in diagnosing these conditions.

While information on some infectious diseases, such as HIV, malaria, and tuberculosis, can be collected through biological assays or cultures, such an equivalent does not exist for certain NCDs. The diagnostic criteria for a condition such as chronic obstructive pulmonary disease (COPD), for example, require medical resources such as spirometry or medical knowledge to interpret FEV1/FVC ratios and differentiate COPD from asthma based on subtle differences in clinical signs and symptoms. Moreover, medical diagnostic tests for NCDs, when existent, are frequently more expensive than for infectious diseases, so surveys tend not to include them; for example, a rapid diagnostic test for malaria costs less than $1 USD in most countries [2], whereas the costs to obtain and measure liver function tests or conduct a 12-lead electrocardiogram are much higher.

Cheaper tests, such as blood pressure and height and weight, are included in many more surveys than the more expensive ones like lipids or blood sugar. Despite these challenges of measuring NCDs, epidemiological studies endeavor to measure the prevalence of conditions, such as asthma [3], depression [4,5], rheumatoid arthritis [6], and COPD [7], in many areas of the developing world. However, there are still significant data gaps and the methods used in these studies have not always been validated against a gold standard clinical and pathological diagnosis. Thus, given that NCDs contribute significantly to the global burden of disease, and given that the diagnosis of NCDs requires clinical expertise and medical resources, the analytic question in this study is whether self-reported signs and symptoms in a questionnaire survey can be accurately assessed by data-driven computational models in order to better measure the burden of these diseases.

Using computers to aid in medical diagnosis has been discussed and explored for decades, ranging from early articles that examined medical “indices” [8], the possibility of diagnostic “machines” [9,10], and the statistics that would feed into diagnostic models [11]. More recently, computational techniques have been implemented for clinical tasks including data-based tasks such as identifying arrhythmias in electrocardiogram tracings and interpretation of computed tomography scans, symptom-based tasks such as predicting maternal morbidity [12], diagnosing asthma [13], and assessing common complaints in an emergency department [14], and for patient-focused tasks, e.g., symptom-based questionnaires such as WebMD and SymCat.

For epidemiological purposes, certain studies measure disease burden based on questionnaire responses, for example, the World Health Survey [15]. Diagnostic classification-given questionnaire-based signs and symptoms have been tested in verbal autopsy (VA) research. In VA studies, interviewers ask family members questions about the signs and symptoms leading up to a death that occurred in the household, and physicians or computer models can be used to classify the estimated cause of death. The advent of advanced computer techniques for solving this problem are described elsewhere [16-22], including a comparison of the performance of different methods and physician-certified VA [23]. This research has provided compelling evidence that computer algorithms can match or outperform physician-certified VA. Although this finding refers to deaths occurred in hospital settings, and further research to test external validity may be required, it bodes well for the idea that computers can also be used for diagnosing NCDs in the living.

This study has two main objectives; first, to develop a questionnaire based on self-reported signs and symptoms and apply an automated technique to estimate the prevalence of NCDs in low-resource settings and, second, to assess the performance of both the questionnaire and the analytical technique at the individual and population level. We refer to this method as “Symptomatic Diagnosis” (SD).

## Methods

### Overview of study design

The SD study consisted of two components: data collection and model validation. The data collection portion consisted of identifying cases of different NCDs in a hospital and then conducting a questionnaire with the patient at a later date. The data were collected in Mexico as part of the Population Health Metrics Research Consortium (PHMRC) Study [22]. The PHMRC Study is an offshoot of the Gates Grand Challenge 13 PHMRC Project, an international collaborative focused on developing better ways to measure health. The model validation component used this validation data to test different approaches to the analytical question of interest.

The SD study developed a questionnaire that focused on 10 NCDs, namely angina pectoris (ICD-10 I20.9), rheumatoid arthritis (ICD-10 M05-M06), cataracts (ICD-10 H25-H26, H28, Q12.0), asthma (ICD-10 J45), COPD (ICD-10 J40-J44, J47), symptomatic cirrhosis (ICD-10 K70.3, K71.7, K74), vision loss (ICD-10 H54), hearing loss (ICD-10 H90-H91), depression (ICD-10 F32, F33), and osteoarthritis (ICD-10 M15-M19, M47). These causes were chosen since they contribute considerably to the burden of disease in Mexico, and because current methods for collecting prevalence data on these conditions are expensive and time-consuming. This questionnaire also collected socio-demographic information. The questionnaire was adapted from the World Health Survey [15] and the PHMRC Household Survey [24]. Information on the signs and symptoms of the respondent is collected, but the questionnaire also asks questions that relate to the respondent’s experience, if any, with health care providers (health care experience; HCE). These questions ask about whether the respondent has ever been diagnosed with different conditions, and whether certain medical procedures or protocols have occurred. The list of items relevant to ascertain the presence of a disease in the questionnaire and HCE indicators is provided in Additional file 1.

### Procedure

Data collection involved three stages: identifying cases for the 10 conditions of interest, identifying controls who did not suffer from any of the NCDs, and then implementing the SD questionnaire at the household of each case and each control.

### Cases

A team of trained coders located approximately 1,200 cases (120 of each of the morbid conditions under study) in 11 public hospitals in the Mexico City area and 120 cases of depression from a psychiatric hospital.

For each condition, a case was defined to be a patient that a physician had diagnosed with the condition and who met a specific set of gold standard diagnosis criteria that was decided on by the PHMRC team. A gold standard diagnosis refers to diagnosis of a specific disease with the highest level of accuracy possible. This involves checking that the diagnosis is based on positive results from a laboratory test or appropriate cabinet and/or checking that the recording and documentation of the appropriate symptoms of the disease were observed during the development of clinical records. To be acceptable, the symptoms of the disease must be observed or documented in a medical record by a physician. The gold standard criteria for each condition are provided in Additional file 2.

We only included cases living in Mexico City who had an address that was identifiable through the hospital records. Once the cases were identified, an interviewer visited each household to administer a SD questionnaire to the cases.

### Controls

We located a population of controls from the records of the Automated Detection and Diagnosis Clinic (CLIDDA) in Mexico City. CLIDDA performs a battery of diagnostic tests on people who are affiliated with the Instituto de Seguridad y Servicios Sociales para los Trabajadores del Estado. We defined a control to be someone who attended the CLIDDA in the last 6 months prior to the data collection, was diagnosed as not having any history of the morbid conditions being studied, was within a similar sex distribution and age range as the cases, was living in the urban area of Mexico City, and whose address was locatable from the CLIDDA records. Individuals with an obvious other disease were not included. We identified a sample of 240 controls. Once the controls were identified by trained coders from the CLIDDA records, appointments were made, and an interviewer visited each household to administer an SD questionnaire.

Signed informed consent was obtained prior to each interview. The project was approved by the institutional review board of the University of Washington and by the research, ethics, and biosafety committees of the National Institute of Public Health, Mexico, and participant institutions.

### Processing

The SD dataset was processed into a format usable by statistical models using the same protocol as described in the PHMRC VA study [22]. Specifically, the duration or continuous survey items are converted to a dichotomous “long duration” item using a median absolute deviation estimator, where the item is considered to be endorsed if it is greater than the long duration cutoff. Cutoffs used in this study are presented in Additional file 3. Categorical items are expanded into being separate dichotomous items for each level or category of the item. For the purpose of clarity, the term “feature” will be used to refer to the dichotomized (endorsed versus not endorsed) items or information used by the model/estimation process, while the term “cause” will be used to refer to “condition” or “illness” or to healthy controls.

### Natural language processing

The SD dataset is composed almost entirely of structured questionnaire items, but free response and text transcription items are also included. One question on the survey asked the interviewer to transcribe text found on any drug containers in the household, and the second free response item asked the interviewer to write down any other pertinent information about the interview that he/she felt was useful.

We implemented techniques based on text mining and natural language processing to capture the “free response” information [25-29]. We were interested in identifying text signals that held some diagnostic value and then in “tokenizing” the free text into data features that could be used by computational algorithms. For example, for the text feature “alcohol”, an interview would have a 1 if that word appeared in the free response section and a 0 if it did not. In addition, some words or expressions are essentially synonymous for data classification purposes (for example, “alcohol”, “alcoholism”, and “alcoholic”). In a process called stemming, we treat the root of the word (in this case, “alcohol”) as the actual text feature instead of the entire word itself. To take care of misspellings, mistranslations, or variations in medical terminology we utilized the dictionary developed for VA analysis that mapped roughly synonymous words to a single text feature. We utilized in this process the TM package in R [30].

### Train-test environment

A critical component of developing and validating data classification models is constructing an appropriate validation environment. A given model must be “trained” on a randomly selected portion of the dataset and then “tested” on an uncontaminated separate portion of the dataset. In this study we split the entire dataset into 75% train data and 25% test data, where the components are sampled by the outcome variable (in this case, the disease). Thus, if there were 100 cirrhosis cases in the full dataset, then 25 would be sampled into the test data and 75 into the train data. This train-test split is repeated 500 times to conduct 500 simulations and to estimate uncertainty around the predictive validity estimates.

Previous research in VA has shown that i) predictive validity is artificially enhanced when test and train composition are similar and ii) the estimated performance of a method is largely a function of the cause composition of the test dataset [31]. To solve the second problem, following Murray et al. [31], we varied the composition of the test data by resampling with replacement based on an uninformative Dirichlet distribution.

### Models

Four data-driven models for VA classification were tested and validated as part of the PHMRC study. We were able to adapt each of these models for use in the SD analysis. Three of the models – Tariff [18], Simplified Symptom Pattern [32], and Random Forest [16] – are capable of diagnosing individual subjects and estimating cause-specific mortality fractions (CSMFs), or in our case, cause-specific prevalence fractions (CSPF), while the King-Lu [33] algorithm can only estimate CSMFs (or in our case CSPFs). The selected method for this analysis was Tariff, which has shown a good performance in previous studies [23] and is described in detail.

Tariff is a simple additive algorithm that uses the calculation of a “tariff” (similar to a Z-score) for each cause-feature combination in the training data followed by a summation and ranking function to predict the most likely causes for each subject in the test dataset. The Tariff for a given cause-feature combination quantifies how uniquely and strongly predictive a given data feature is for a given cause. The Tariff for cause *i* and feature *j* is calculated as:

$$ Tarif{f}_{ij}=\frac{x_{ij}\kern0.5em -\kern0.5em  Median\kern0.5em \left({x}_j\right)}{Interquartile\kern0.5em  Range\kern0.5em {x}_j} $$

where Tariff_ij_ is the tariff for cause i, feature j, x_ij_ is the fraction of subjects with cause i for which there is a positive response for item j, median (x_j_) is the median fraction with a positive response for feature j across all causes, and interquartile range x_j_ is the interquartile range of positive response rates for feature j averaged across causes.

For each subject in the SD dataset, we compute summed Tariff scores for each cause:

$$ Tariff\kern0.5em  Scor{e}_{ki}=\sum_{j\kern0.5em =\kern0.5em 1}^w\kern0.5em  Tarif{f}_{ij}{x}_{jk} $$

The Tariff scores for each cause are ranked across all subjects, and the top-ranked cause for each subject is assigned as the diagnosis for that subject.

### Analysis

We assessed and compared the capability of the SD model using the VA performance metrics described by Murray et al. [31]. SD is capable of i) predicting whether or not an individual suffers from different NCDs and ii) estimating the fraction of individuals in a population who suffer from a given condition. Consequently, the performance of SD should be quantified in both the individual and population domains.

### Chance-corrected concordance

Chance-corrected concordance (CCC) is a measure of a method’s ability to correctly diagnose a condition in an individual. However, because random assignment of N different causes would be correct 1/N times, this metric must also be adjusted for random chance [31].

The formal calculation of CCC for cause j (CCCj) is:

$$ CC{C}_j=\frac{\left(\frac{T{P}_j}{T{P}_j+F{N}_j}\right)-\left(\frac{1}{N}\right)}{1-\left(\frac{1}{N}\right)} $$

where TP is true positives, FN is false negatives, and N is the number of causes or conditions (11 in this study).

### Cause-specific prevalence fraction (CSPF) accuracy

Following Murray et al. [31], we used CSPF accuracy as a metric to assess the ability of the questionnaire to estimate prevalence fractions, analog to CSMF in a verbal autopsy study. In our case, CSPF accuracy, which is an aggregate measure across all causes k, is formally defined as:

$$ CSPFAccuracy=1-\frac{{\displaystyle \sum_{j=1}^k\left|CSP{F}_j^t-CSP{F}_j^{\Pr ed}\right|}}{2\left(1-Min\left(CSP{F}_j^t\right)\right)} $$

Where the superscript for CSPF refers to true (“t”) or predicted (“pred”) cause fractions. The denominator reflects the maximum possible CSPF error in the given test split:

$$ \mathrm{CSPF}\ \mathrm{Maximum}\ \mathrm{Error} = 2\left(1\hbox{-} \mathrm{Minimum}\ \left(\mathrm{C}\mathrm{S}\mathrm{P}{{\mathrm{F}}_{\mathrm{j}}}^{\mathrm{t}}\right)\right) $$

Hence, CSPF accuracy can be described as 1 minus the sum of absolute errors divided by the maximum error. A CSPF accuracy of 1 would indicate perfect cause fraction predictions, while 0 would indicate the worst possible model.

We assessed the performance of SD in the two metrics described above and in terms of cause fraction absolute error, which allows for inspection of its performance in measuring prevalence fractions for each cause. Each type of validation was conducted across 500 splits of data. We tested each method under two conditions: with all data features and with all data features excluding HCE information.

We also analyzed whether SD methods systematically over- or underestimate the prevalence fractions. Using the true and estimated prevalence fractions from 500 splits, we conducted linear regressions where the estimated prevalence fraction was a function of the true prevalence. Stata, R, and Python were used for all analysis and data management.

## Results

### Symptomatic diagnosis interviews

We collected 1,379 questionnaires with an accompanying gold standard diagnosis. The number of SD interviews conducted for each of the 10 conditions and for controls are provided in Table 1. This table also provides the age and sex distribution by condition and shows that the project gathered approximately the target number of interviews or more for each condition and for the control group.

**Table 1:** **Characteristics of the study participants for each condition**

**Condition**	**Number of interviews**	**Mean age**	**Standard deviation age**	**% Male**
**Angina pectoris**	107	62.7	11.9	69%
**Asthma**	117	42.7	12.8	26%
**Chronic obstructive pulmonary disease**	108	69.1	11.0	43%
**Cataracts**	108	68.1	13.4	38%
**Cirrhosis**	104	51.4	11.3	81%
**Depression**	100	39.4	15.2	30%
**Hearing loss**	205	47.3	9.1	29%
**Osteoarthritis**	107	62.1	11.5	20%
**Rheumatoid arthritis**	119	52.1	12.3	8%
**Vision loss**	106	54.9	16.8	39%
**Control**	198	40.3	6.0	14%

Although questionnaires were analyzed using all of the different methods mentioned above, this section presents only the results derived from the Tariff method. Results from analysis using other methods are presented in Additional file 4 as robustness checks.

Table 2 provides the mean CCC for the Tariff method across 500 splits, with and without HCE. Overall, CCC increases with HCE. We calculated the estimated and true prevalence cause fractions for each test split of data. These true and estimated cause fractions were used to calculate absolute errors and CSPF accuracy across 500 splits. Table 2 also provides the median CSPF accuracy for the Tariff method across 500 splits, with and without HCE information. As in the case of CCC, accuracy increased with the inclusion of HCE information.

**Table 2:** **Mean chance-corrected concordance and median cause-specific prevalence fraction accuracy across causes including uncertainty intervals, with and without health care experience (HCE), using the Tariff method**

	**Chance-corrected concordance (CCC)**		**Cause-specific mortality fractions accuracy**	
	**CCC estimate**	**95% uncertainty interval**	**CCC estimate**	**95%** **uncertainty interval**
**No HCE**	53.4%	(53.2–53.9%)	0.772	(0.765–0.779)
**With HCE**	66.1%	(65.6–66.5%)	0.826	(0.818–0.834)

**Figure 1:**
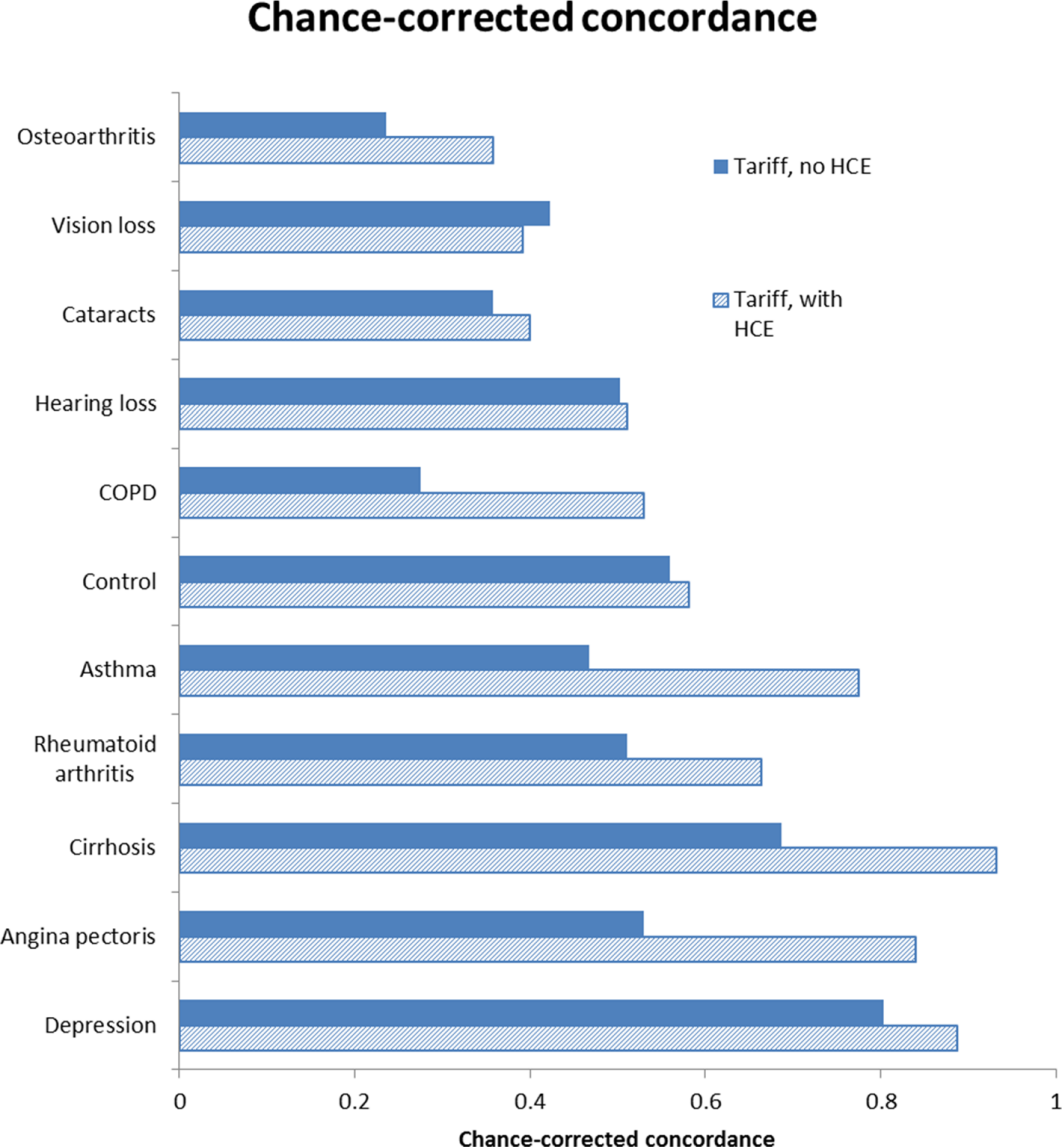
**Cause-specific chance-corrected concordance with and without health care experience.**

**Figure 2:**
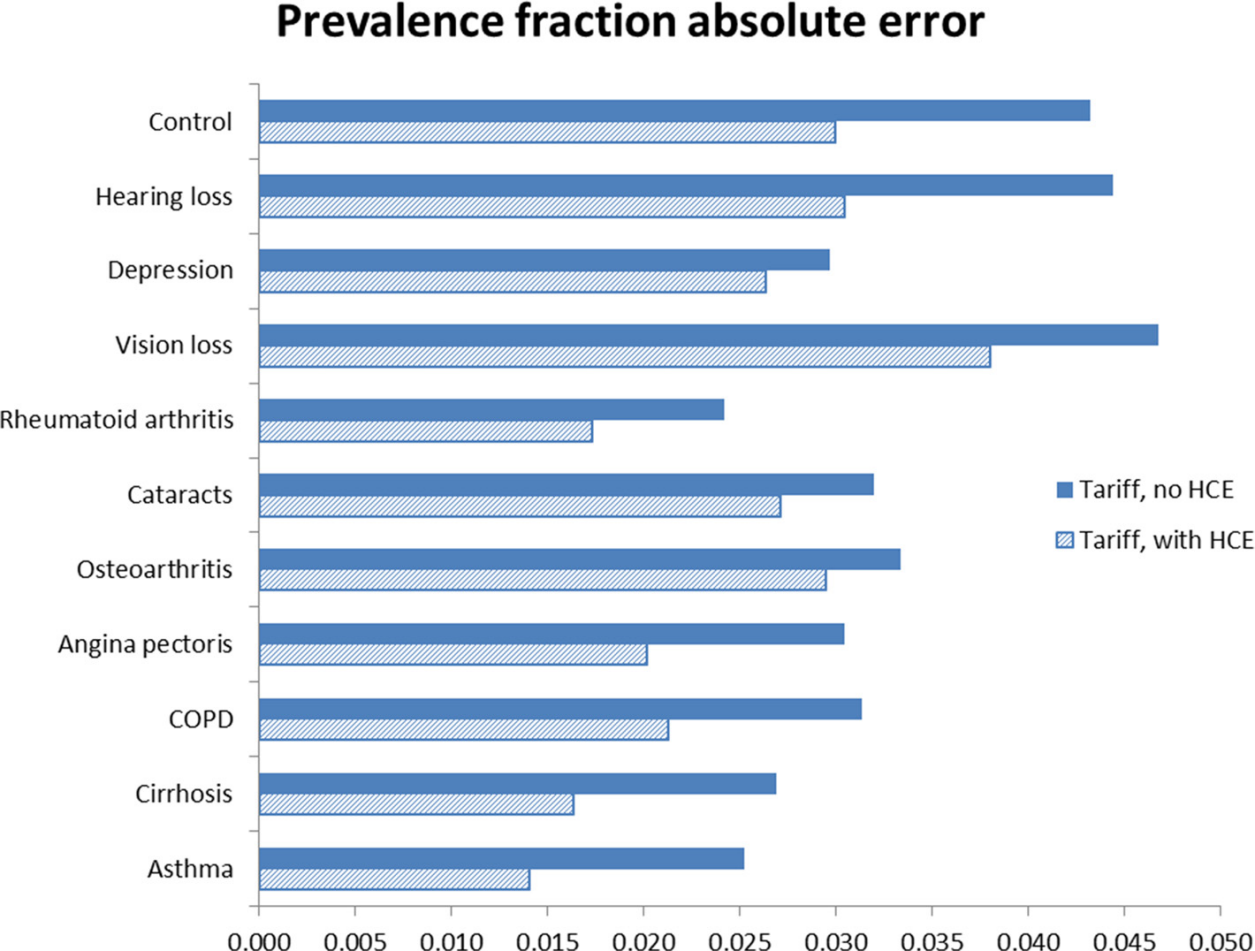
**Cause-specific prevalence fraction absolute errors with and without health care experience.**

Figures 1 and 2 show CCC and prevalence fraction absolute errors for each specific condition with and without HCE. We observed that depression has high CCC regardless of whether HCE information is used, while vision loss, cataracts, and osteoarthritis experience lower performance. Some causes, such as asthma, rheumatoid arthritis, cirrhosis, or angina, increase their CCC more than others by the inclusion of HCE information. Prevalence fraction absolute errors are higher in the analysis without HCE, and are smaller for causes like rheumatoid arthritis, asthma, and cirrhosis.

**Figure 3:**
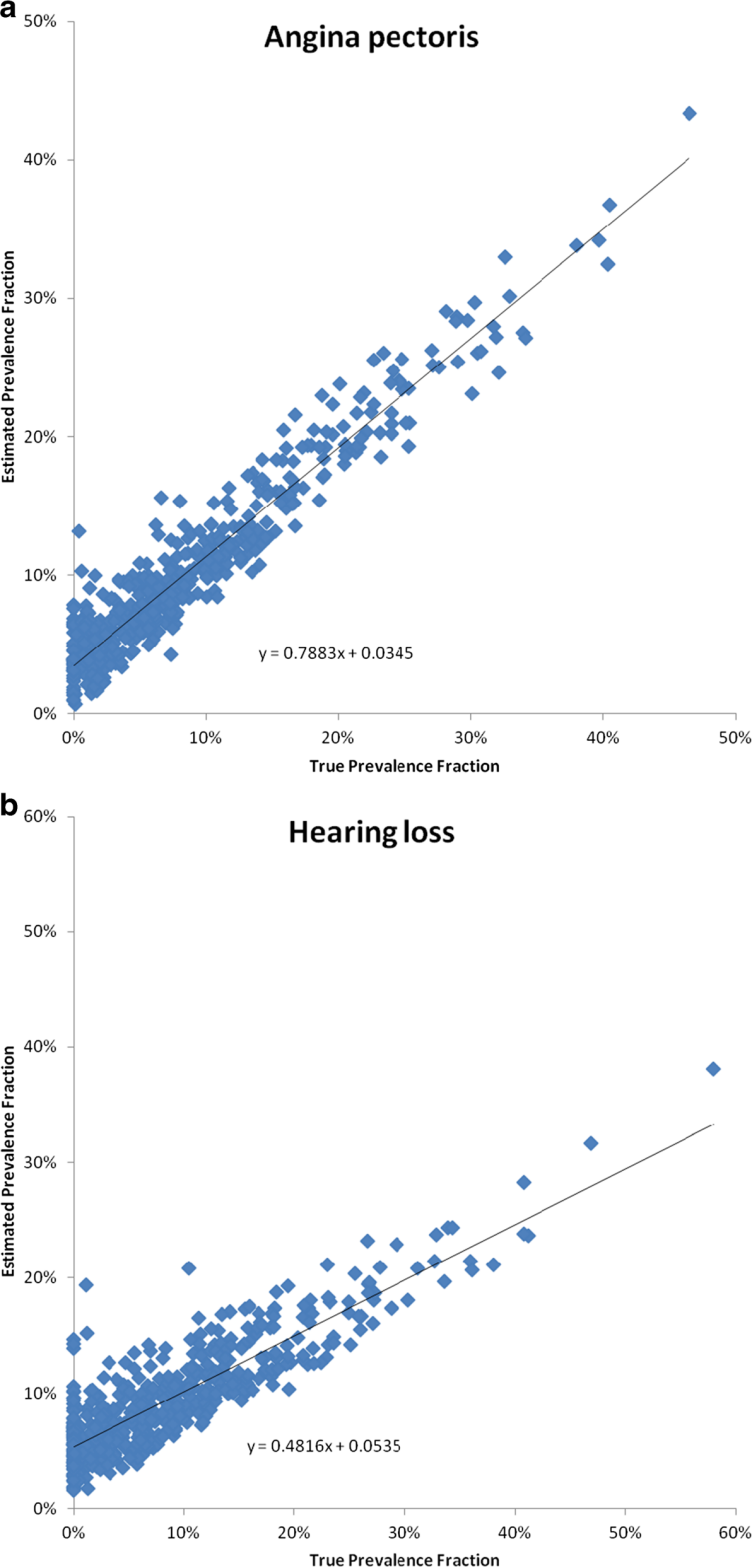
**True and estimated prevalence fractions using the Tariff Method with health care experience for 500 splits for (a) angina pectoris and (b) hearing loss.**

In the analysis to check whether SD systematically over- or underestimates the prevalence fractions, we conducted linear regressions where the estimated prevalence fraction was a function of the true one. An illustration of this analysis for angina pectoris with HCE is provided in Figure 3a. This figure and associated coefficient and intercept illustrate how the SD for this cause tends to slightly underestimate the prevalence of angina pectoris, except for very low true prevalence fractions. In contrast, the equivalent scatterplot in Figure 3b for hearing loss shows more overestimation when the true prevalence fraction is 0 but a general systematic underestimation for larger prevalence fractions.

### Cross-classification and cause aggregation

We found that the SD achieved high CCC for the causes of angina pectoris, depression, and cirrhosis. However, vision loss and osteoarthritis experienced lower performance. To explore if there was some cross-classification between vision loss with cataracts due to the similar clinical presentation of these conditions, we used cross-classification or “confusion” matrices. An example of a confusion matrix for a single split (prior to undergoing the Dirichlet-based resampling) is shown in Additional file 5. This confusion matrix shows how 8 out of 24 true vision loss cases were correctly classified as vision loss, but 8 were misclassified as cataracts. Out of 27 true cataracts cases, 10 were correctly classified as cataracts, but 5 were misclassified as vision loss.

This investigation of cause assignments indicated that there could be considerable cross-classification but also that the features most strongly associated with vision loss or cataracts had a much weaker association than other feature-cause combinations. This suggests the possibility of increasing performance by combining similar causes. So, instead of differentiating between vision loss and cataracts, we generated a combined category of “vision loss or cataracts.” We measured the effect of this aggregation on performance and found that overall CCC increased by approximately 3% in absolute terms and that CSPF accuracy increased by 0.032 in absolute terms (Table 3). The prevalence fraction absolute error and CSPF accuracy for the nine-cause aggregation is shown in Additional file 6.

**Table 3:** **Chance-corrected concordance and cause-specific prevalence fraction accuracy for nine-cause aggregation using the Tariff Method, with and without health care experience (HCE)**

	**Chance-corrected concordance (%)**	
	**No HCE (%)**	**With HCE (%)**
Cirrhosis	82.4	90.6
Depression	87.5	88.7
Angina pectoris	80.1	84.1
Asthma	68.9	76.4
Arthritis	63.4	70.3
Vision loss or cataracts	57.0	57.4
Chronic obstructive pulmonary disease	41.6	49.5
Hearing loss	47.1	47.9
Control	56.5	57.8
Mean chance-corrected concordance (%)	65.0	69.2
Median cause-specific prevalence fractions accuracy	0.842	0.858

## Discussion

The PHMRC SD study presents a novel source of data and an innovative application of VA research to computational estimation of NCD burden. The study identified cases of 10 NCDs that had been diagnosed with gold standard criteria and then conducted a questionnaire with over 100 patients for each condition. The questionnaire was designed to estimate prevalence using data-driven methods, specifically the Tariff method, which has been validated in VA studies. By applying Tariff to the PHMRC dataset, we sought to demonstrate that the questionnaire-based approach of SD may be a valuable asset to future epidemiological research concerning the burden of NCDs. We further simulated the application of Tariff in the field by testing the performance with the inclusion/exclusion of HCE information, which allowed us to determine the viability of using Tariff in areas with no health care, and by testing performance in samples of test data with random cause compositions. The results of the study can thus be considered robust due to i) the gold-standard validation of the questionnaire responses, ii) the calculation of predictive validity in various test data compositions, and iii) comparisons with the performance of current epidemiological measurement approaches.

We observed that the Tariff method applied to the SD questionnaire is a promising approach for collecting prevalence data on the NCDs outlined in this study. Tariff was capable of estimating prevalence fractions within 3% for each condition. Additionally, 5 of the 11 conditions have a CCC of over 60%. Estimation of prevalence fractions within 3% for these chronic conditions would allow for higher resolution epidemiological information in areas with sparse data, and for the conditions with high performance in terms of CCC, this technology could be used for diagnoses in the field without requiring extensive medical expertise or other tools or resources.

While Tariff demonstrated accurate results in measuring certain conditions, it was found to be less accurate in others. The lower performance of Tariff in identifying vision loss, cataracts, and osteoarthritis can be explained partially through cross-classification error, but the relatively poor performance of hearing loss with Tariff may be a surprising result since it seems reasonable that hearing loss patients would express somewhat salient signs and symptoms compared with other conditions. The analysis of endorsement rates of all features by true cause showed that one of the obstacles in diagnosing hearing loss based on this questionnaire derives from a lack of items with a high endorsement rate for hearing loss but not for other causes. For example, one item asks, “Have you ever had your hearing checked by a provider?” While the endorsement rates for hearing loss are high (91.2%) for this item, cases with the other conditions also endorse this item at a fairly high frequency (82.2% of the controls and 71.8% of the asthma cases). In contrast, causes that had better performance tend to have an item that has a high endorsement rate for that cause but not for others. For example, the question “Have you had a period lasting several days when you lost interest in most things that you usually enjoy such as pastimes, relationships, or work?” has a 90% endorsement rate among depression cases, but no higher than 30.3% endorsement for other causes.

In our literature-based comparison to current methods, we observed that Tariff was capable of matching or outperforming the questionnaire-based methods such as the Rose questionnaire and the composite international diagnostic interview (CIDI) depression questions in terms of prevalence estimation (Table 4). The SD questionnaire essentially includes the Rose questionnaire, CIDI depression questions, and the World Health Survey asthma questions. Since, in theory, the positive endorsement of the items for these questions should essentially ensure correct diagnosis, it may be surprising that the data-driven SD methods can achieve higher performance than these current approaches. That is, we did not need to “tell” Tariff that endorsement of the Rose questions indicates angina pectoris; rather, Tariff determined those relationships on its own. The result in some ways parallels the finding in VA research that computational methods can compete with and possibly outperform physician-certified autopsy.

**Table 4:** **Absolute errors in prevalence estimates from SD method to literature-based approaches**

**Condition**	**Test**	**Absolute error (95% CI)**	**Symptomatic diagnosis-Tariff method with health care experience absolute error (95% CI)**
Asthma	World Health Survey (WHS): Doctor (MD) diagnosis (Dx) [34]	0.023 (0.020, 0.025)	0.014 (0.012, 0.016)
	WHS MD Dx OR asthma medications (Rx) [34]	0.023 (0.020, 0.025)	
	WHS MD Dx OR asthma Rx OR wheezing/whistling attacks [34]	0.092 (0.087, 0.095)	
Angina pectoris	Rose questionnaire [35,36]	0.082 (0.073, 0.088)	0.020 (0.018, 0.022)
Depression	Composite international diagnostic interview questionnaire [37]	0.059 (0.054, 0.064)	0.016 (0.015, 0.017)

Current estimation of COPD, rheumatoid arthritis, osteoarthritis, vision loss, hearing loss, cirrhosis, and cataracts can be undertaken with a high level of accuracy in a clinical setting, but their diagnosis requires specialized diagnostic equipment and extensive medical training. Certainly, it is desirable to collect the most accurate information possible, but access to these tools and resources is not possible in all areas of the world. Furthermore, SD has the unique advantage of being able to collect information on every condition after conducting a single interview. The alternative approaches of carrying diagnostic equipment for each of these conditions or conducting multiple cause-specific diagnostic surveys in parallel seems very implausible. As discussed in the introduction, this is an unfortunate paradox since the areas lacking these resources are also likely the areas that have the worst health. SD, in this regard, is a valuable alternative to collecting more refined information in a resource-poor setting. A household survey can be conducted virtually anywhere in the world. Currently, Demographic and Health Surveys and World Health Surveys cover areas of the world such as Sudan, Cote d’Ivoire, and Democratic Republic of Congo, for example. If access to the tools and expertise to diagnose these conditions in these areas is unavailable, then SD-based epidemiology could be a practical alternative. The use of SD methods in low-resource or inaccessible areas to identify and focus attention on the chronic disease burden could also help address the aforementioned paradox. Furthermore, training field workers to conduct an SD survey seems likely to be less expensive than making available the resources to diagnose all of the conditions outlined in this study. Cumulatively, this flexibility makes SD a compelling alternative strategy for measuring the burden of these conditions.

### Limitations

Our study had some inherent limitations. One of the main limitations and questions in VA research is that questionnaire responses for deaths that occur in the community could be systematically different than the responses from deaths that occur in hospitals. If the response patterns are sufficiently different, then the computational methods could perform differently than expected when they are implemented in the field. However, this limitation, which applies also to SD, is essentially a normative question. It is not possible to develop data-driven models unless this limitation is accepted, and as previous research in VA has shown, data-driven models can match or outperform expert-based models [23]. To deal with this limitation, we drew 500 samples with different prevalence of NCDs under study from our study sample, and attempted to simulate cases where the respondent did not have access to health care by conducting analyses in which we withheld HCE features.

A second limitation is that the study did not include individuals whose address could not be found, and this may potentially introduce a bias if individuals who were not found are systematically different in their socioeconomic status or other characteristics from the ones who were located. The main reason for not locating cases was that the patient provided the address of a relative to have access to some hospitals in Mexico City; therefore, we do not think that it is likely that there are clear differences in the socioeconomic status of these individuals. However, it is possible that the study may exclude some individuals from lower socioeconomic status that could not be located.

Since prevalence data are sparse in many areas of the world, it is important to consider the potential implementation of the SD methods outlined in this study in countries besides Mexico. The 10 NCDs considered in this study are also highly prevalent in areas of Africa and Asia, and this consideration raises the question if there exist systematic cultural variations in questionnaire response data. It seems plausible that response patterns for something as sensitive as a medical interview will have cultural idiosyncrasies. This limitation can be addressed by further collection of validated SD questionnaire responses in other countries. In fact, additional validated SD questionnaire response data would strengthen the performance of the existing models. Furthermore, the computational SD methods can readily be retrained on any further validation data collected, though similar to VA, the general SD approach will be strongest if a central data repository is maintained such that the central Tariff matrix can be continually updated.

Finally, while the inclusion of healthy controls is generally a strength in this study, it is possible that the healthy controls can be ill with minor conditions. The inclusion of these controls is important, however, because it allows for a model to predict that a person does not suffer from a given condition despite possibly presenting some of the signs and symptoms associated with that condition. For example, 50% of the controls report a non-productive cough, which is not a dramatically lower endorsement rate than asthma, in which 58% of cases reported a non-productive cough. The inclusion of controls highlights one of the important differences between SD and VA: every person who dies has an underlying cause of death, but not every living person has an underlying illness. There were also some differences in the characteristics of the health controls compared to the rest of the study participants as shown on Table 1; specifically, the controls tend to be slightly younger and are more frequently female than the other participants.

### Future implementation

The central question for future implementation of SD methods is whether this method provides adequate accuracy and usability to be used for epidemiologic data collection. Certainly, in the hierarchy of epidemiological data, self-reported signs and symptoms in an interview setting has historically been considered relatively lower-quality data; however, one of the important findings in this study was that the methods and criteria used in traditional epidemiologic studies of these diseases was in fact less accurate than the methods tested here. Thus, this study provides promising evidence that self-reported signs and symptoms combined with techniques such as the Tariff method may be more valuable than previously understood. Regardless, it will be important to further validate the performance of SD methods, particularly by using further cross-validation with data collected from outside this study. Similarly, while this study focused on selected NCDs in one country, it will be important to conduct further research both in other countries and with other conditions in order to generalize the capabilities of SD. This will be particularly important when it comes to NCDs with more protean clinical presentations, in areas where it is more difficult to collect data, and in patients who may have multiple comorbidities. Ideally, future work will further develop the capabilities of SD in assessing the prevalence of other NCDs in other areas of the world, contributing to a better understanding of the burden of NCDs globally.

With these considerations, the implementation of the tool developed in this study requires two further steps for interested users. The first step is facilitating the implementation of the questionnaire itself. The questionnaire is provided in this study and can hypothetically be used as-is. However, current work in VA is moving toward using tablet devices that can use questionnaire software such as Open Data Kit Collect to facilitate data collection. Options such as geotagging, digital imagery, and improved instrument clarity/organization make this an even more compelling data collection approach. The second required step is developing a user-friendly software package that readily conducts the method described in this study. Reducing these barriers will facilitate more rapid use of the methods outlined in this study to improve the collection of health information for NCDs.

## Conclusions

The SD study had the goal to develop better instruments and methods for measuring population health, particularly in resource-poor settings where clinicians are not available to assist in diagnosis of NCDs. To this end, the study was a success in that it found that the Tariff method could accurately measure the prevalence of several important conditions. This study provides a promising way to improve strategies for population health measurement and to produce instruments that are scientific, standardized, and widely applicable across different resource-poor settings. Although more work is required to test this method in other NCDs and in different settings, the SD questionnaire combined with the Tariff method, has the potential to help researchers better measure the burden of NCDs and to additionally enable policymakers and researchers to help address persistent inequities in health outcomes in both the developed and the developing world.

## Additional files

Additional file 1:
**Medically relevant symptomatic diagnosis questionnaire items.**
Additional file 2:
**Gold standard criteria** **[** 38 **].**
Additional file 3:
**Cutoff for endorsement of each continuous/duration questionnaire item.**
Additional file 4:
**Results of analysis using different analytical methods.**
Additional file 5:
**Example of confusion matrix for true and estimated cause classifications.**
Additional file 6:
**Prevalence fraction absolute error and CSPF accuracy for nine-cause Tariff.** Method aggregation, with and without HCE.

## Abbreviations

CCC: Chance-corrected concordance
CIDI: Composite international diagnostic interview
CLIDDA: Automated detection and diagnosis clinic
COPD: Chronic obstructive pulmonary disease
CSMF: Cause-specific mortality fractions
CSPF: Cause-specific prevalence fractions
HCE: Health care experience
NCD: Non-communicable disease
PHMRC: Population health metrics research consortium
SD: Symptomatic diagnosis
VA: Verbal autopsy
