# Supplementary material for: Validating estimates of prevalence of non-communicable diseases based on household surveys: the symptomatic diagnosis study
Source: BMC Med. 2015 Jan 26;13:15. doi: 10.1186/s12916-014-0245-8 (PMC4306245; doi:10.1186/s12916-014-0245-8)
Supplement: Additional file 1: — Medically relevant symptomatic diagnosis questionnaire items. [file 12916_2014_245_MOESM1_ESM.pdf]

Additional file 1: Medically relevant symptomatic diagnosis questionnaire items.

Item	Question *(HCE items are italicized)*
**sd4_01**	*Have you ever been told by a health provider that you have chronic bronchitis, emphysema, or chronic obstructive pulmonary disease (COPD)?*
**sd4_02a**	*How long ago, in months or years, were you told by a health provider that you have chronic bronchitis, emphysema, or chronic obstructive pulmonary disease (COPD)? [specify]*
**sd4_02b**	*How long ago, in months or years, were you told by a health provider that you have chronic bronchitis, emphysema, or chronic obstructive pulmonary disease (COPD)?*
**sd4_03**	*Are you currently taking medication for chronic bronchitis, emphysema, or chronic obstructive pulmonary disease (COPD)?*
**sd4_04**	*Have you ever been told by a health provider that you have heart failure?*
**sd4_05a**	*How long ago, in months or years, were you told by a health provider that you have heart failure?*
**sd4_52b**	*How long ago, in months or years, were you told by a health provider that you have heart failure?*
**sd4_06**	*Are you currently taking medication for heart failure?*
**sd4_07**	*Have you ever been told by a health provider that you have cirrhosis?*
**sd4_8a**	*How long ago, in months or years, were you told by a health provider that you have cirrhosis?*
**sd4_8b**	*How long ago, in months or years, were you told by a health provider that you have cirrhosis?*
**sd4_09**	*Are you currently taking medication for cirrhosis?*
**sd4_10**	*Have you ever been told by a health provider that you have liver failure?*
**sd4_11a**	*How long ago, in months or years, were you told by a health provider that you have liver failure?*
**sd4_11b**	*How long ago, in months or years, were you told by a health provider that you have liver failure?*
**sd4_12**	*Are you currently taking medication for liver failure?*
**sd4_13**	*Have you ever been told by a health provider that you have angina?*
**sd4_14a**	*How long ago, in months or years, were you told by a health provider that you have angina?*
**sd4_14b**	*How long ago, in months or years, were you told by a health provider that you have angina?*
**sd4_15**	*Are you currently taking medication for angina?*
**sd4_16**	*Have you ever been told by a health provider that you have angina?*
**sd4_17**	*What type of arthritis did they say you had?*
**sd4_18a**	*How long ago, in months or years, were you told by a health provider that you have arthritis?*
**sd4_18b**	*How long ago, in months or years, were you told by a health provider that you have arthritis?*
**sd4_19**	*Are you currently taking medication for arthritis?*

**sd4_20**	*Have you ever been told by a health provider that you have asthma?*
**sd4_21a**	*How long ago, in months or years, were you told by a health provider that you have asthma?*
**sd4_21b**	*How long ago, in months or years, were you told by a health provider that you have asthma?*
**sd4_22**	*Are you currently taking medication for asthma?*
**sd4_23**	*Have you ever been told by a health provider that you have depression?*
**sd4_24a**	*How long ago, in months or years, were you told by a health provider that you have depression?*
**sd4_24b**	*How long ago, in months or years, were you told by a health provider that you have depression?*
**sd4_25**	*Are you currently taking medication for depression?*
**sd4_26**	*Have you ever been told by a health provider, including an optician, that you have a cataract in one or both of your eyes (that is, an opacity in the lens of the eye)?*
**sd4_27**	*Have you ever had eye surgery to remove your cataract(s)?*
**sd4_28**	*Have you ever had your eyes checked by a health provider, including an optician?*
**sd4_29a**	*How long ago, in months or years, was your vision checked by a health provider?*
**sd4_29b**	*How long ago, in months or years, was your vision checked by a health provider?*
**sd4_30**	Do you wear glasses or contact lenses?
**sd4_31**	Wearing your glasses or contact lenses, how much difficulty do you have in seeing and recognizing a person you know across the road (i.e. from a distance of about 20 meters)?
**sd4_32**	Wearing your glasses or contact lenses, how much difficulty do you have in seeing and recognizing an object at arm's length or in reading?
**sd4_33**	If you are NOT wearing glasses or contact lenses, how much difficulty do you have in seeing and recognizing a person you know across the road (i.e. from a distance of about 20 meters)?
**sd4_34**	If you are NOT wearing your glasses or contact lenses, how much difficulty do you have in seeing and recognizing an object at arm's length or in reading?
**sd4_35**	Does respondent use glasses/contacts or not (for skip pattern)
**sd4_36**	How much difficulty do you have in seeing and recognizing a person you know across the road (i.e. from a distance of about 20 meters)?
**sd4_37**	How much difficulty do you have in seeing and recognizing an object at arm's length or in reading?
**sd4_38**	*Have you ever had your hearing checked by a health provider?*
**sd4_39a**	*How long ago, in months or years, was your hearing last checked by a health provider?*
**sd4_39b**	*How long ago, in months or years, was your hearing last checked by a health provider?*
**sd4_40**	Do you have deafness or trouble hearing in one or both ears without the help of a hearing aid?
**sd4_41**	*Are you currently wearing a hearing aid?*
**sd4_42**	*Do you have trouble hearing in one or both ears even with a hearing aid?*
**sd5_1**	Have you had productive cough for at least two weeks in a year especially in the cold seasons?
**sd5_2**	Have you had shortness of breath during the time you had a productive cough?
**sd5_3**	Have you had non-productive cough?
**sd5_4**	Have you had attacks of shortness of breath?
**sd5_5**	Have you had shortness of breath that gets worse when you lie down, like during sleep?

**sd5_6**	Have you had wheezing?
**sd5_7**	Have you had chest pain?
**sd5_8**	Have you had swelling around your ankle?
**sd5_9**	Have you had a period lasting several days when you felt sad, empty or depressed?
**sd5_10**	Did this period last more than 2 weeks?
**sd5_11**	Did this period last most of the day?
**sd5_12**	Was this period nearly every day?
**sd5_13**	During this period, did your appetite increase or decrease?
**sd5_14**	During this period, did you lose or gain weight without it being your intention?
**sd5_15**	During this period did you notice any slowing down in your thinking?
**sd5_16**	During this period, did you have insomnia or sleep excessively most of the time?
**sd5_17**	During this period, did you feel tired and without energy all of the time?
**sd5_18**	During this period, did you feel guilty or useless?
**sd5_19**	During this period, did you have trouble concentrating or making decisions?
**sd5_20**	During this period, did you want to hurt yourself or be dead, or did you think of how to kill yourself or commit suicide?
**sd5_21**	Have you had a period lasting several days when you lost interest in most things that you usually enjoy such as pastimes, relationships, or work?
**sd5_22**	Did this period last more than 2 weeks?
**sd5_23**	Did this period last most of the day?
**sd5_24**	Was this period nearly every day?
**sd5_25**	During this period, did your appetite increase or decrease?
**sd5_26**	During this period, did you lose or gain weight without it being your intention?
**sd5_27**	During this period did you notice any slowing down in your thinking?
**sd5_28**	During this period, did you have insomnia or sleep excessively most of the time?
**sd5_29**	During this period, did you feel guilty or useless?
**sd5_30**	During this period, did you have trouble concentrating or making decisions?
**sd5_31**	During this period, did you want to hurt yourself or be dead, or did you think of how to kill yourself or commit suicide?
**sd5_32**	During this period, did you feel tired and without energy all of the time?
**sd5_33**	During this period, did those depressive symptoms cause significant discomfort or make it difficult to work or socialize, or affect your life in general in any other way?
**sd5_34**	During this period, were those symptoms caused completely by the loss of a loved one?
**sd5_35**	During this period, were those symptoms similar to those that someone in similar circumstances would experience?
**sd5_36**	During this period, do you remember having taken any medicine or drug right before or associated with the start of those depressive symptoms?
**sd5_37**	During this period, do you remember having suffered from or acquired an illness just before or associated with the beginning of those depressive symptoms?
**sd5_38**	Do you know if any family member such as your daughter, son, mother, father, grandfather or grandmother suffered from or were treated for depression at any point?
**sd5_39**	Do you have difficulty following a conversation in a noisy environment?
**sd5_40**	Are you able to hear out of both of your ears?

**sd5_41**	Are you able to hear when you are using a phone?
**sd5_42**	Do you have ringing in the ears?
**sd5_43**	Have you ever experienced back pain (including disc problems) during the last 30 days?
**sd5_44a**	How many days did you have this back pain for during the last 30 days? [specify]
**sd5_44b**	How many days did you have this back pain for during the last 30 days?
**sd5_45**	Have you experienced joint inflammation in a symmetrical pattern (both sides of the joint affected rather than just one side)?
	During the last 12 months, have you experienced pain, aching, stiffness or swelling in or around the joint which was not related to an injury and lasted for more than a month?
**sd5_46a**	...A (neck)
**sd5_46b**	...B (right shoulder)
**sd5_46c**	...C (left shoulder)
**sd5_46d**	...D (right elbow)
**sd5_46e**	...E (left elbow)
**sd5_46f**	...F (right hand)
**sd5_46g**	...G (left hand)
**sd5_46h**	...H (right hip)
**sd5_46i**	...I (left hip)
**sd5_46j**	...J (right knee)
**sd5_46k**	...K (left knee)
**sd5_46l**	...L (right ankle)
**sd5_46m**	...M (left ankle)
**sd5_46n**	...N (left foot)
**sd5_46o**	...O (right foot)
**sd5_46p**	...P (wrist)
**sd5_46q**	...Q (thumb)
**sd5_46r**	...R (pinkie finger, lower joint)
**sd5_46s**	...S (ring finger, lower joint)
**sd5_46t**	...T (middle finger, lower joint)
**sd5_46u**	...U (index finger, lower joint)
**sd5_46v**	...V (pinkie finger, upper joint)
**sd5_46w**	...W (index finger, upper joint)
**sd5_46x**	...X (ring finger, upper joint)
**sd5_46y**	...Y (middle finger, upper joint)
**sd5_47**	Stiffness in the joint in the morning after getting up from bed, or after a long rest of the joint without movement?
**sd5_48**	How long does this stiffness last?
**sd5_49**	Does this stiffness go away after exercise or movement in the joint?
**sd5_50**	Have you experienced attacks of wheezing or whistling breathing?
**sd5_51**	Attack of wheezing that came on after you stopped exercising or some other physical activity?
**sd5_52**	A feeling of tightness in your chest?

**sd5_53**	Waking up with a feeling of tightness in your chest in the morning or any other time?
**sd5_54**	An attack of shortness of breath that came on without obvious cause when you were not exercising or doing some physical activity?
**sd5_55**	Pain or discomfort in your chest when you walk uphill or hurry?
**sd5_56**	Pain or discomfort in your chest when you walk at an ordinary pace on level ground?
**sd5_57**	Chest discomfort or pain for skip pattern
**sd5_58**	What do you do if you get the pain or discomfort when you are walking?
**sd5_59**	If you stand still, what happens to the pain or discomfort? Is it…
	Will you show me where you usually experience the pain or discomfort?
**sd5_60a**	…A: right shoulder
**sd5_60b**	…B: right side chest
**sd5_60c**	…C: neck area
**sd5_60d**	…D: upper middle chest
**sd5_60e**	…E: lower middle chest
**sd5_60f**	…F: left side chest
**sd5_60g**	…G: left shoulder
**sd5_60h**	…H: abdomen
**sd5_61**	In the past 12 months have you experienced cloudy or blurry vision?
**sd5_62**	Vision problems with light, such as seeing glare from bright lights, or seeing halos around lights?
**sd5_63**	Have you noticed, or has anyone told you that you have whiteness in your eye?
**sd5_64**	Have you passed dark urine during the past two weeks (dark yellow or plain tea color)?
**sd5_65**	Did you have icterus (yellow tinge in your body, especially the conjunctiva, palms and skin) during the past two weeks?
**sd5_66**	Was your skin itchy during the past two weeks?
**sd5_67**	Did you have malena (dark brown or black stools) during the past two weeks?
**sd5_68**	Have you ever vomited blood (haematemesis)?
**sd5_69**	Have you noticed abdominal enlargement during the past two weeks?
**sd5_70**	Did you notice swelling around your ankles during the past two weeks?
**sd5_71**	*Have you ever had hepatitis in your life or has a diagnosis of hepatitis ever been made by a health provider?*
**sd6_1**	*Do you have any medicines in the house that a health provider has prescribed for you or given to you?*
**sd6_2**	*May I see what medicines you personally have been using in the last 2 weeks?*
**sd6_3**	*Name of prescription from bottle/label:*
**sd6_4a**	*How frequently do you use this medicine?*
**sd6_4b**	*How frequently do you use this medicine?*
**sd6_5**	*Name of prescription from bottle/label:*
**sd6_6a**	*How frequently do you use this medicine?*
**sd6_6b**	*How frequently do you use this medicine?*
**sd6_7**	*Name of prescription from bottle/label:*
**sd6_8a**	*How frequently do you use this medicine?*
**sd6_8b**	*How frequently do you use this medicine?*

**sd6_9**	*Name of prescription from bottle/label:*
**sd6_10a**	*How frequently do you use this medicine?*
**sd6_10b**	*How frequently do you use this medicine?*
**sd6_11**	*Name of prescription from bottle/label:*
**sd6_12a**	*How frequently do you use this medicine?*
**sd6_12b**	*How frequently do you use this medicine?*
**sd7_1**	*Is there anything the research team should be aware of about this respondent that may have affected the quality of data?*
