# Supplementary material for: Validating estimates of prevalence of non-communicable diseases based on household surveys: the symptomatic diagnosis study
Source: BMC Med. 2015 Jan 26;13:15. doi: 10.1186/s12916-014-0245-8 (PMC4306245; doi:10.1186/s12916-014-0245-8)
Supplement: Additional file 2: — Gold standard criteria [ 38 ]. [file 12916_2014_245_MOESM2_ESM.pdf]

Additional file 2. Gold Standard criteria

Angina pectoris

Gold standard cases for angina pectoris required chest pain when doing physical exertion or when they feel strong emotions. The pain must have been relieved by rest and must be validated by one of the first three tests and a chest radiograph:

- Test 1: Resting electrocardiogram: QRS-segment deviation, Q waves and ST segment changes and T waves, or
- Test 2: Electrocardiogram with exercise or stress test: QRS segment deviation, Q waves, and ST segment changes and T waves, or
- Test 3: Resting electrocardiogram during chest pain: QRS segment deviation, Q waves and ST segment changes and T waves
- Chest x-ray in patients with signs or symptoms of congestive heart failure, valve disease, heart disease, pericardial disease, or aortic dissection/aneurysm.

Cataract

Gold standard cataract cases required the opacity of the lens to be confirmed by a slit lamp examination. This could also include cataract with retinopathy.

Symptomatic cirrhosis

The gold standard cases were required to have four of the following results of liver function tests:

- Anemia (detected on a complete blood count)
- Abnormalities of coagulation
- Elevated liver enzymes
- Elevated bilirubin
- Low serum albumin
- Enlarged liver (seen on an abdominal radiograph)
- ALT (alanine aminotransferase) > 2.1

Test	Men	Women
ALT (IU/L)	10 – 40	8 – 35

- AST (aspartate aminotransferase) > 2

Test	Men	Women	Children
AST (IU/L)	20 – 40	15 – 30	Newborn: 25 – 75
			Baby: 15 – 60

- ALP (alkaline phosphatase)

Test	Men	Women
ALP (IU/L)	50 – 120	50 – 120

- Prothrombin

Test	Men	Women
Prothrombin time (seconds)	> 30	> 30

- Albumin

Test	Men	Women	Children
Albumin (g/dL)	1-35 years: 3.5-4.8	1-35 years: 3.5-4.8	0-1 years: 2.9 – 5.5
Albumin: globulin > 1	Reduced in > 40 years	Reduced in > 40 years	

- GGT (gamma-glutamyl transpepsidase) > 2

Test	Men	Women
GGT (IU/L)	2 – 30	1 – 24

Decrease or loss of hearing

Gold standard cases for hearing loss must have met one of the first two test-based requirements, and then must have undergone a hearing test:

1. The inability to hear a whisper, normal speech, and the ticking of a clock, or
2. The inability to hear a tuning fork through air and hear the pitch of the bone.

In a hearing test with detailed audiometry, patient must not be able to hear tones from 250 Hz - 8,000 Hz at 25 dB or lower.

Osteoarthritis

Gold standard cases for osteoarthritis required all of the following elements:

- Pain in the large joints (particularly knees),
- Swelling and limited movement,
- Radiography showing loss of joint cartilage, narrowing of joint space between adjacent bones, the formation of bone spurs, and decreased joint space and spicules.

Rheumatoid arthritis

Gold standard cases for rheumatoid arthritis required four of the following:

- Stiffness in the morning,
- Arthritis in three or more joint areas,
- Arthritis in the joints of the hands,
- Symmetrical arthritis,
- Rheumatoid nodules,
- Blood serum rheumatoid factor,
- Radiographic changes typical of arthritis.

Asthma

The gold standard cases for asthma required spirometry showing reduced forced expiratory volume in 1 second (FEV1), reduced ratio of FEV1 to forced vital capacity (FVC), and reduced peak expiratory flow (PEF).

Chronic obstructive pulmonary disease (COPD)

Gold standard cases for COPD required all of the following:

- Spirometry: FEV1/FVC < 0.7
- Spirometry: FEV1 ≥ 80% predicted

Decrease or loss of visual acuity

Gold standard cases required a visual acuity test with one of the following results:

1. Mild visual disability (farsightedness):
   Visual acuity in the eye to see better than 6/10 to 6/18 (20/32 to 20/63 inclusive)
2. Moderate visual disability (farsightedness):
   Visual acuity in the eye to see better than 6/24 to 6/48 (20/80 to 20/160 inclusive)
3. Severe visual disability (farsightedness):
   Visual acuity in the eye to see better than 6/60 to 3/60 (20/200 to 20/400 inclusive)
4. Visual impairment deep (farsightedness):
   Visual acuity in the better eye to see 2/60 (= 20/500 to 20/1000 inclusive)
5. Near to blindness (farsightedness):
   Visual acuity in the better eye to see 1/60 or worse (= worse than 20/1000 inclusive)
6. Near visual disabilities:
   Near binocular visual acuity worse than 6/10 (= 20/32) and distant visual acuity in the eye to see better than 6/7.5 (20/25) or better

Depression

The study protocol for depression cases in the SD study followed a different format than the other conditions. Upon a patient's arrival to the Instituto Nacional de Psiquiatría, a third-year psychiatry resident applied the Mini-International Neuropsychiatric Interview (MINI) questionnaire and diagnosed cases as depressed using the Diagnostic and Statistical Manual of Mental Disorders, 4th edition (DSM-IV) criteria [12]. Gold standard cases were required to meet the following criteria:

- Criterion A: The presence of at least five of the following symptoms for at least two weeks. One of the symptoms must be the first (sad mood or anhedonia):

  - Sad mood, dysphoric or irritable most of the day, almost every day, as reported by the individual,
  - Anhedonia or diminished ability to enjoy or show interest and/or pleasure in usual activities, most of the day, almost every day,
  - Decrease or increase in weight or appetite almost every day,
  - Insomnia or hypersomnia, almost every day,
  - Psychomotor agitation or retardation almost every day (big enough to be observed by others, not just feelings of agitation or retardation),
  - Asthenia, almost every day,
  - Recurrent feelings of worthlessness or guilt, almost every day (not only self-blaming for the fact of being sick),
  - Decreased ability or intellectual ability, almost every day (either a subjective attribution or an observation by another person)
  - Recurrent thoughts of death or suicidal (not only fear of death), recurrent suicidal thoughts without a specific plan or suicide attempt.
- Criterion B: There are no signs for mixed affective disorders (manic and depressive), schizoaffective disorder, or schizophrenia disorders.
- Criterion C: The symptoms have a negative impact on the social, occupational, or other vital areas of the patient.
- Criterion D: The symptoms are not explained by the consumption of toxic substances or drugs, or by another illness.
- Criterion E: The symptoms are not explained by a reaction of grief at the loss of an important person in the patient's life, the symptoms remain for more than 2 months or indicate functional inability, morbid inutility concerns, suicidal ideation, psychotic symptoms or psychomotor retardation.
