# Supplementary material for: Validating estimates of prevalence of non-communicable diseases based on household surveys: the symptomatic diagnosis study
Source: BMC Med. 2015 Jan 26;13:15. doi: 10.1186/s12916-014-0245-8 (PMC4306245; doi:10.1186/s12916-014-0245-8)
Supplement: Additional file 3: — Cutoff for endorsement of each continuous/duration questionnaire item. [file 12916_2014_245_MOESM3_ESM.pdf]

Additional file 3. Cutoff for endorsement of each continuous/duration questionnaire item.

Item	Question	Cutoff
**sd4_02b**	How long ago, in months or years, were you told by a health provider that you have chronic bronchitis, emphysema, or chronic obstructive pulmonary disease (COPD)?	9.25 years
**sd4_05a**	How long ago, in months or years, were you told by a health provider that you have heart failure?	1 year
**sd4_11b**	How long ago, in months or years, were you told by a health provider that you have liver failure?	5.25 years
**sd4_14b**	How long ago, in months or years, were you told by a health provider that you have angina?	4.7 years
**sd4_18b**	How long ago, in months or years, were you told by a health provider that you have arthritis?	9.75 years
**sd4_21b**	How long ago, in months or years, were you told by a health provider that you have asthma?	17.75 years
**sd4_24b**	How long ago, in months or years, were you told by a health provider that you have depression?	5.4 years
**sd4_29b**	How long ago, in months or years, was your vision checked by a health provider?	3 years
**sd4_39b**	How long ago, in months or years, was your hearing last checked by a health provider?	3.4 years
