# Supplementary material for: Validating estimates of prevalence of non-communicable diseases based on household surveys: the symptomatic diagnosis study
Source: BMC Med. 2015 Jan 26;13:15. doi: 10.1186/s12916-014-0245-8 (PMC4306245; doi:10.1186/s12916-014-0245-8)
Supplement: Additional file 4: — Results of analysis using different analytical methods. [file 12916_2014_245_MOESM4_ESM.pdf]

Additional file 4. Results of analysis using different analytic methods

Table 1: Mean chance-corrected concordance across causes including uncertainty intervals, calculated as the median across 500 splits for each method, with and without HCE.

Method	HCE	Chance-corrected concordance (95% CI)
**Tariff**	no HCE	53.4% (53.2%, 53.9%)
	with HCE	66.1% (65.6%, 66.5%)
**SSP**	no HCE	53.9% (53.5%, 54.3%)
	with HCE	67.9% (67.4%, 68.3%)
**Random Forest**	no HCE	52.4% (52.1%, 52.7%)
	with HCE	66.9% (66.5%, 67.3%)

Table 2: Cause-specific chance-corrected concordance using different analytic methods, calculated as the median across 500 splits for each method, with and without HCE.

	**CCC Tariff (%)**		**CCC SSP (%)**		**CCC RF (%)**	
	no HCE	*with HCE*	*no HCE*	*with HCE*	*no HCE*	*with HCE*
**Depression**	80.3	88.7	85.1	87.6	79.8	84.6
**Angina pectoris**	52.9	83.9	67.6	93.6	61.0	87.1
**Cirrhosis**	68.6	93.2	69.7	91.3	67.5	93.3
**Rheumatoid arthritis**	51.1	66.4	55.0	90.4	43.4	66.8
**Asthma**	46.7	77.5	60.7	87.8	52.2	81.7
**COPD**	27.5	52.9	28.3	56.5	35.8	64.4
**Hearing loss**	50.3	51.1	50.9	52.8	42.2	47.0
**Cataracts**	35.8	40.0	38.1	57.9	37.4	48.4
**Vision loss**	42.2	39.1	20.3	18.4	27.8	33.1
**Osteoarthritis**	23.6	35.8	13.0	25.9	29.6	40.1
**Control**	56.0	58.1	50.4	51.4	45.0	48.7

Table 3: Median CSPF accuracy using different analytic methods with and without HCE information, including uncertainty intervals, calculated as the median across 500 splits for each method.

Method	HCE	CSPF accuracy (95% UI)
**Tariff**	no HCE	0.772 (0.765, 0.779)
	with HCE	0.826 (0.818, 0.834)
**SSP**	no HCE	0.764 (0.753, 0.771)
	with HCE	0.817 (0.809, 0.828)
**Random Forest**	no HCE	0.775 (0.767, 0.781)
	with HCE	0.822 (0.814, 0.829)
**King-Lu**	no HCE	0.736 (0.729, 0.745)
	with HCE	0.765 (0.758, 0.772)

Table 4: Cause-specific prevalence fraction absolute error by analytical method, with and without HCE*.

Prevalence fraction absolute error	Tariff		SSP		RF		KL	
	no HCE	*with HCE*	*no HCE*	*with HCE*	*no HCE*	*with HCE*	*no HCE*	*with HCE*
Asthma	0.025	0.014	0.034	0.016	0.031	0.015	0.038	0.033
Cirrhosis	0.027	0.016	0.016	0.007	0.021	0.015	0.042	0.036
COPD	0.031	0.021	0.034	0.024	0.034	0.023	0.048	0.043
Angina pectoris	0.030	0.020	0.026	0.024	0.032	0.026	0.044	0.039
Osteoarthritis	0.033	0.029	0.039	0.031	0.038	0.032	0.044	0.043
Cataracts	0.032	0.027	0.032	0.031	0.036	0.029	0.045	0.042
Rheumatoid arthritis	0.024	0.017	0.037	0.029	0.031	0.019	0.037	0.031
Vision loss	0.047	0.038	0.036	0.030	0.049	0.037	0.043	0.043
Depression	0.030	0.026	0.059	0.026	0.032	0.021	0.016	0.016
Hearing loss	0.044	0.030	0.032	0.028	0.032	0.033	0.038	0.034
Control	0.043	0.030	0.029	0.025	0.030	0.027	0.030	0.029

*This table is displayed as a heat map, where larger errors are shaded in darker reds and smaller errors are darker greens. Unshaded (white) is medium error.

Figure 1: Cause-specific chance-corrected concordance using different analytic methods with and without HCE.

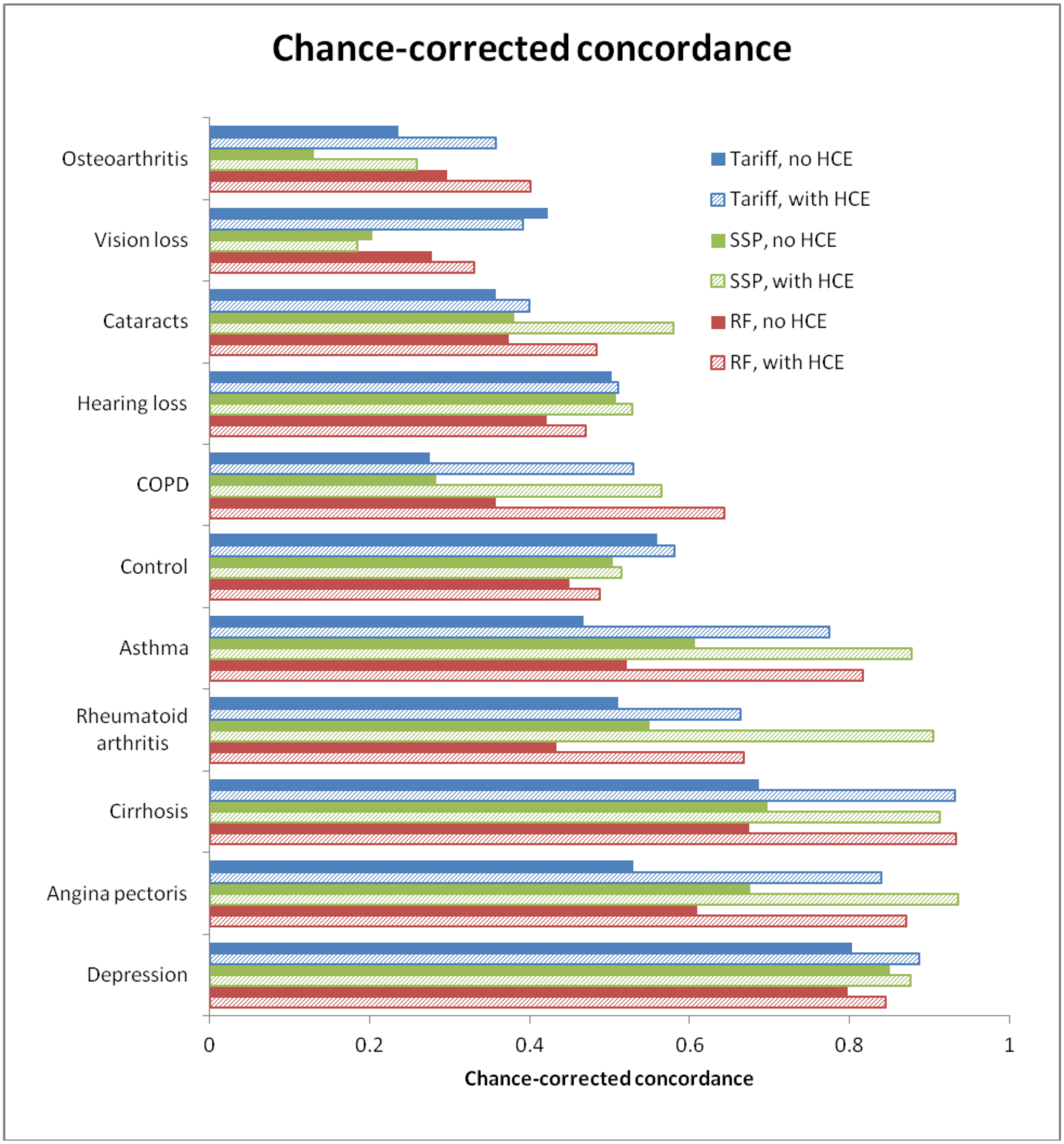

Figure 2: Cause-specific prevalence fraction error using different analytic methods of analysis, with and without HCE.

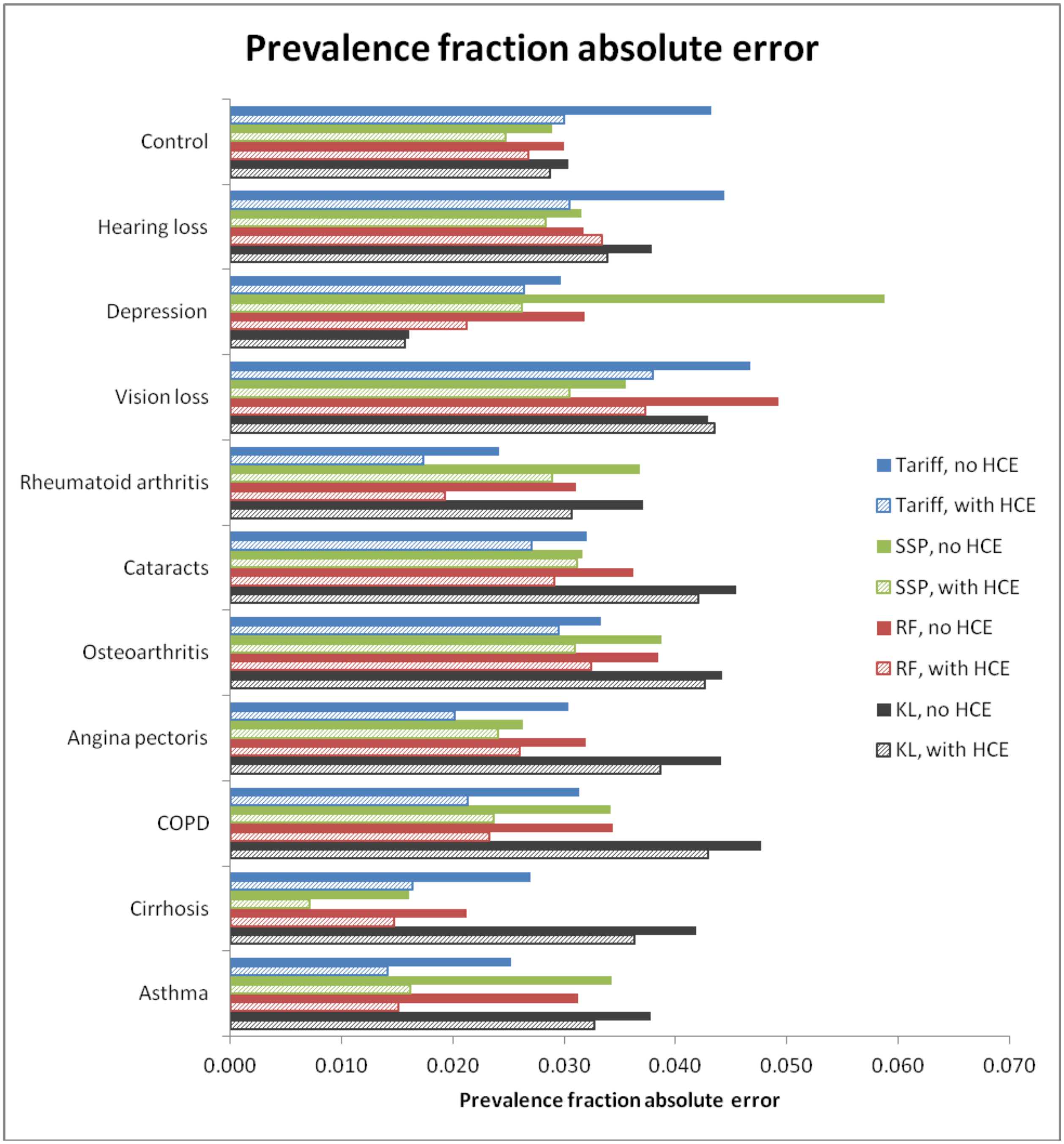

Additional file 5. Example of confusion matrix for true and estimated cause classifications.

True cause: / Predicted cause:	Angina pectoris	Osteoarthritis	Rheumatoid arthritis	Asthma	Cataracts	COPD	Cirrhosis	Depression	Hearing loss	Vision loss	Control	**Total**
Angina pectoris	24	1	0	0	0	0	0	0	1	0	1	**27**
Osteoarthritis	2	8	6	0	4	0	0	1	2	0	1	**24**
Rheumatoid arthritis	1	4	19	0	1	0	1	2	0	0	0	**28**
Asthma	0	1	0	15	0	1	0	0	1	0	0	**18**
Cataracts	1	1	0	0	10	4	1	2	1	5	2	**27**
COPD	1	0	0	1	1	2	0	0	0	0	0	**5**
Cirrhosis	0	0	0	0	0	0	20	0	0	2	1	**23**
Depression	0	0	0	0	0	0	0	11	0	1	0	**12**
Hearing loss	0	0	0	0	0	0	0	5	5	0	2	**12**
Vision loss	3	0	0	0	8	0	1	1	3	8	0	**24**
Control	2	0	0	3	1	0	0	3	21	0	20	**50**
**Total**	**34**	**15**	**25**	**19**	**25**	**7**	**23**	**25**	**34**	**16**	**27**	**250**

Additional file 6. Prevalence fraction absolute error and CSPF accuracy for nine-cause Tariff Method aggregation, with and without HCE. *

**Prevalence fraction absolute error**	**Estimates**	
	no HCE	*with HCE*
Cirrhosis	0.013	0.013
Asthma	0.017	0.014
COPD	0.025	0.024
Angina pectoris	0.030	0.027
Arthritis	0.035	0.029
Depression	0.032	0.029
Hearing loss	0.035	0.032
Vision loss or cataracts	0.038	0.036
Control	0.036	0.031
CSPF accuracy	0.842	0.858

*This table is displayed as a heat map, where larger error is indicated with darker reds and smaller error is indicated with darker greens.
