# Supplementary material for: Validating estimates of prevalence of non-communicable diseases based on household surveys: the symptomatic diagnosis study
Source: BMC Med. 2015 Jan 26;13:15. doi: 10.1186/s12916-014-0245-8 (PMC4306245; doi:10.1186/s12916-014-0245-8)
Supplement: Additional file 5: — Example of confusion matrix for true and estimated cause classifications. [file 12916_2014_245_MOESM5_ESM.pdf]

Additional file 5. Example of confusion matrix for true and estimated cause classifications.

Predicted cause: / True cause:	Angina pectoris	Osteoarthritis	Rheumatoid arthritis	Asthma	Cataracts	COPD	Cirrhosis	Depression	Hearing loss	Vision loss	Control	**Total**
Angina pectoris	24	1	0	0	0	0	0	0	1	0	1	**27**
Osteoarthritis	2	8	6	0	4	0	0	1	2	0	1	**24**
Rheumatoid arthritis	1	4	19	0	1	0	1	2	0	0	0	**28**
Asthma	0	1	0	15	0	1	0	0	1	0	0	**18**
Cataracts	1	1	0	0	10	4	1	2	1	5	2	**27**
COPD	1	0	0	1	1	2	0	0	0	0	0	**5**
Cirrhosis	0	0	0	0	0	0	20	0	0	2	1	**23**
Depression	0	0	0	0	0	0	0	11	0	1	0	**12**
Hearing loss	0	0	0	0	0	0	0	5	5	0	2	**12**
Vision loss	3	0	0	0	8	0	1	1	3	8	0	**24**
Control	2	0	0	3	1	0	0	3	21	0	20	**50**
**Total**	**34**	**15**	**25**	**19**	**25**	**7**	**23**	**25**	**34**	**16**	**27**	**250**
