# Supplementary material for: Validating estimates of prevalence of non-communicable diseases based on household surveys: the symptomatic diagnosis study
Source: BMC Med. 2015 Jan 26;13:15. doi: 10.1186/s12916-014-0245-8 (PMC4306245; doi:10.1186/s12916-014-0245-8)
Supplement: Additional file 6: — Prevalence fraction absolute error and CSPF accuracy for nine-cause Tariff. Method aggregation, with and without HCE. [file 12916_2014_245_MOESM6_ESM.pdf]

Additional file 6. Prevalence fraction absolute error and CSPF accuracy for nine-cause Tariff Method aggregation, with and without HCE. *

**Prevalence fraction absolute error**	**Estimates**	
	no HCE	*with HCE*
Cirrhosis	0.013	0.013
Asthma	0.017	0.014
COPD	0.025	0.024
Angina pectoris	0.030	0.027
Arthritis	0.035	0.029
Depression	0.032	0.029
Control	0.036	0.031
Hearing loss	0.035	0.032
Vision loss or cataracts	0.038	0.036
CSPF accuracy	0.842	0.858

*This table is displayed as a heat map, where larger error is indicated with darker reds and smaller error is indicated with darker greens.
